# Toward a More Definitive Goldilocks Mastectomy: Simultaneous Addition of the Lateral Intercostal Perforator Flap

**DOI:** 10.1097/GOX.0000000000002132

**Published:** 2019-03-13

**Authors:** Jean-Claude D. Schwartz

**Affiliations:** From the Georgia Breast Surgery, Lawrenceville, Ga.

## Abstract

The Goldilocks mastectomy was originally described as a safe technique that allowed for a single-stage autologous reconstruction in the patient who either was a poor candidate or was not interested in traditional multistage postmastectomy reconstructive approaches. This technique involved a skin-sparing mastectomy through Wise incisions and utilized the residual cutaneous flaps to create a breast mound. The final result was often less than optimal with regards to volume as the surgeon was limited by the amount of residual skin and fat that remained after the mastectomy. The best results were obtained in women with ptosis and an elevated body mass index. Here, we improve on these results by combining the Goldilocks mastectomy and the lateral intercostal artery perforator (LICAP) flap to provide a more definitive, autologous, single-stage, reconstructive option after mastectomy. Fourteen consecutive women underwent simultaneous bilateral Goldilocks mastectomy with bilateral LICAP flap augmentation. All patients successfully completed their reconstruction and were healed by 10 weeks postoperatively. The combination of the Goldilocks mastectomy and LICAP flap provides reconstructive surgeons an opportunity to provide a greater proportion of women a more definitive, safe, single-stage autologous reconstructive option after mastectomy.

## Abstract 2

Supplemental Digital Content is available in the text.

## INTRODUCTION

The Goldilocks mastectomy was devised as a safe, single-stage breast reconstruction for women who were either poor candidates or not interested in the more traditional reconstructive approaches.^1^ Many of these women were obese and/or diabetic with significant macromastia and ptosis, all known risk factors for complications after implant-based and autologous reconstruction.^2–4^ Although the Goldilocks mastectomy offered a safer option in these patients, the reconstructive outcome was often lacking with regards to volume as the residual cutaneous mastectomy flaps after oncological resection were usually insufficient to create a breast mound of good size and proportion.^1^ To address this issue, we sought to add a safe and reliable autologous reconstructive technique that could supplement the Goldilocks mastectomy in the same operative setting.

The lateral intercostal artery perforator (LICAP) flap has been well described in reconstruction after breast-conserving oncological surgery^5^ and in the massive weight loss (MWL) patient.^6^ The donor site heals reliably well regardless of body mass index (BMI), and the flap viability is likely independent of the degree of macromastia or obesity. Here, we describe our experience with 28 breasts in 14 consecutive patients who underwent Goldilocks mastectomy with free nipple grafts with simultaneous addition of the LICAP flap.

### Surgical Technique

**Fig. 1.**
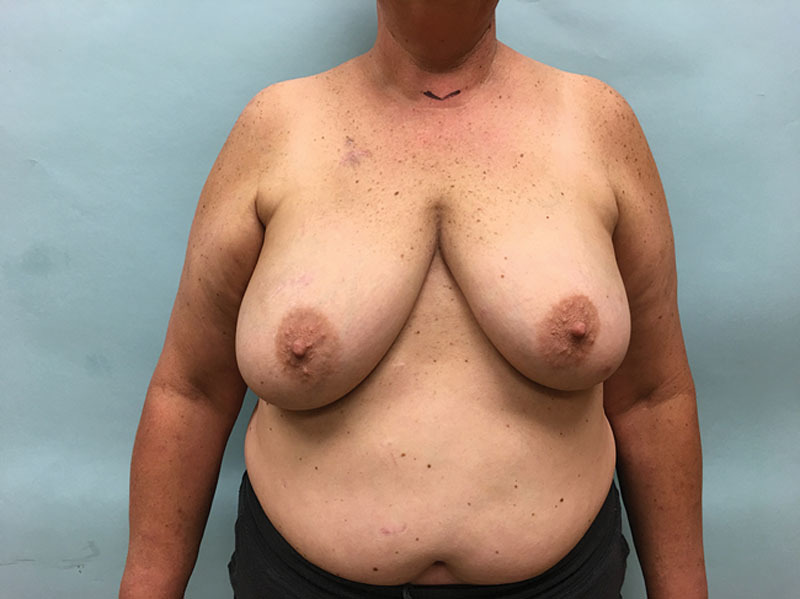
Preoperative photograph of a 51-year-old obese (BMI 32) patient with grade 2 ptosis who demands a single-stage reconstructive procedure. She is wary of regular magnetic resonance imaging to survey the integrity of silicone breast implants and would prefer to never under another surgical procedure after her index operation. She refused immediate bilateral deep inferior epigastric flaps after consultation with a microvascular surgeon. Given her obesity, we feel that the Goldilocks mastectomy with free nipple grafts and LICAP flap is her best reconstructive option. She is shown here 6 weeks after completing chemotherapy for a 2-cm triple negative right breast cancer.

**Fig. 2.**
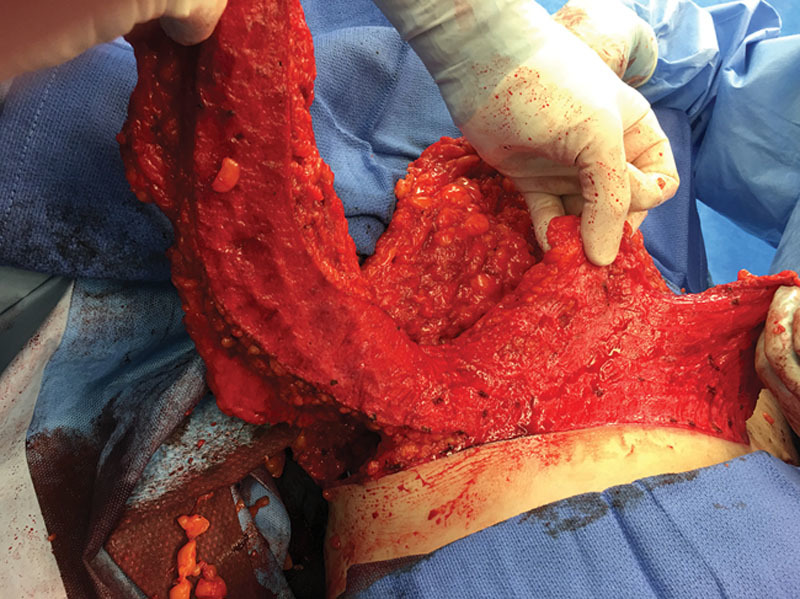
The deepithelialized inferior mastectomy flap and LICAP flap are shown here in continuity. The LICAP flap is turned 180 degrees on its pivot point near the anterior border of the latissimus dorsi muscle and imbricated on itself, centered in the meridian of the breast, secured to the pectoralis muscle to reconstruct a breast mound. The inferior mastectomy flap is then brought over the LICAP flap to provide additional volume and projection. The medial and lateral Wise flaps are then brought down to the inframammary fold in the meridian over the reconstructed breast mound with the intervening tissue between the medial and lateral vertical limbs providing even more projection. After confirmation of a negative subareolar biopsy, the nipple areola complexes are grafted into position.

**Fig. 3.**
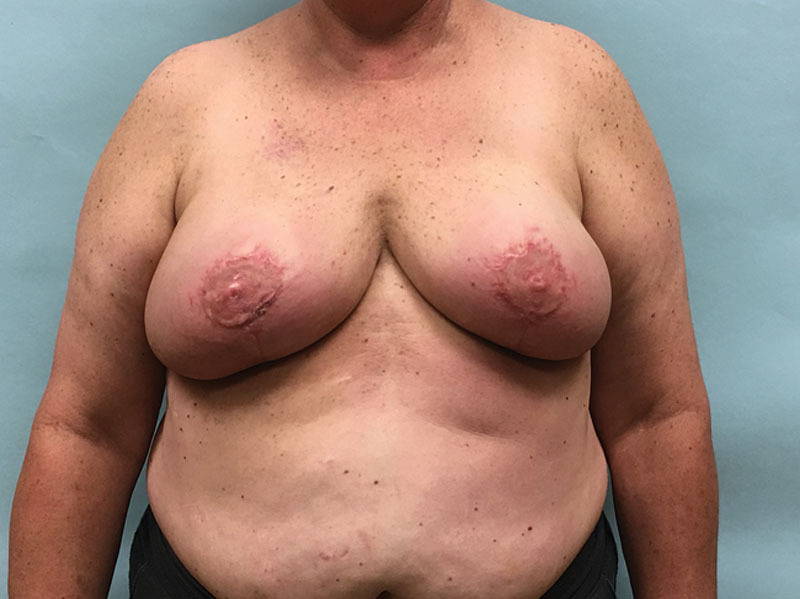
Despite a 6 × 8 cm area of necrosis at the T junction involving the right lateral Wise flap, she heals within 9 weeks with daily dressing changes and without major aesthetic sequelae. She has excellent symmetry and has completed her cancer resection and reconstruction in a single surgery that takes 275 minutes. She requires no further adjuvant treatment nor surgical intervention. She is discharged home on the day of surgery.

**Fig. 4.**
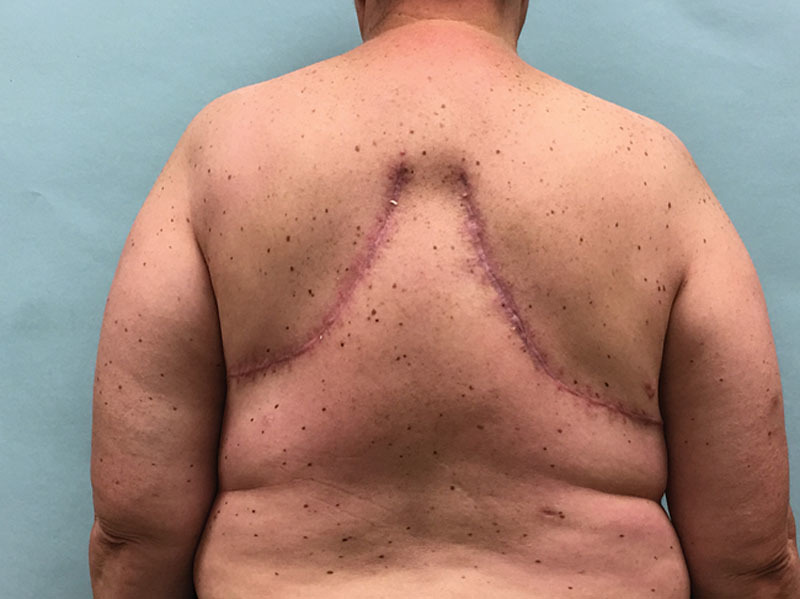
The extended LICAP flap donor sites are demonstrated which heal reliably well. In the obese patient, they can provide a significant amount of additional needed volume to the reconstructed breast. There are no functional deficits or chronic pain as the underlying muscles are left undisturbed.

Patients are marked using the standard Wise pattern in preparation for the Goldilocks mastectomy as previously described.^1^ We excluded all poorly controlled diabetics (hemoglobin A1C > 7). Active smokers were required to abstain for 4 weeks before surgery and for an additional 4 weeks postprocedure. A representative obese, high-risk patient who refuses implant-based reconstruction is shown in Figure 1. In addition to the Wise pattern, markings for the extended LICAP flap were designed as previously described.^7^ Patients are initially placed in the prone position and bilateral LICAP flaps are raised. The patient is then placed supine and the mastectomy is performed and sentinel lymph nodes evaluated through a horizontal incision that divides the breast transversely in 2, connecting the medial and lateral extensions from the vertical limbs to the inframammary fold. The nipple areola complex is excised and used as a free nipple graft. The inferior mastectomy flap is deepithelialized in continuity with the LICAP flap (Fig. 2). We then proceeded with mobilization of the LICAP flap 180 degrees on its pivot point at the anterior border of the latissimus where the perforators arise and translate the flap as far medially as possible to create a breast mound. The inferior mastectomy flap is then wrapped around the LICAP flap to provide more volume and projection. The breast is then closed in standard Wise fashion followed by free nipple grafting. The entire procedure is planned and performed by a single surgeon, which is critical to the feasibility of alternating between the oncological resection and reconstructive portions of this procedure without delay and would be difficult for 2 independent surgical teams to coordinate efficiently. Figure 3 demonstrates our patient 3 months postoperative and Figure 4 reveals her postoperative donor site scars. We demonstrate 2 additional patients who underwent combined Goldilocks mastectomy and LICAP flap reconstruction [see figure, **Supplemental Digital Content 1**, which displays 67-year-old morbidly obese female (BMI 42) with a remote history of right partial mastectomy and radiotherapy for invasive breast cancer, http://links.lww.com/PRSGO/B17; see figure, **Supplemental Digital Content 2**, which displays preoperative photograph of a 62-year-old female who is mildly overweight (BMI 27) with a stage 1 right breast cancer. Despite this aggressive undermining, these back incisions heal reliably well, http://links.lww.com/PRSGO/B18).

## RESULTS

A total of 14 patients and 28 breasts underwent simultaneous Goldilocks mastectomy and LICAP flap reconstruction. The average BMI of our patients was 33.5 (range 24.4–44) and the average operative time was 275 minutes (range 222–330 minutes) for single-stage oncological resection and reconstruction. Three patients underwent neoadjuvant chemotherapy and 4 underwent adjuvant chemotherapy. Of 28 donor sites, 26 healed without complication. Two donor sites required postoperative wound care. There were no instances of flap loss or necrosis that required reoperation. Six breasts suffered complications from the Wise pattern closure, all of which healed with conservative measures by 10 weeks after surgery. Of 28 breasts, 4 had areas of fat necrosis 6 months postoperatively, none of which required intervention.

## DISCUSSION

The Goldilocks mastectomy provides patients a single-stage, relatively low risk, autologous reconstructive option.^1^ However, the final reconstructed breast volume is limited by the residual cutaneous mastectomy flaps which typically results in a smaller than ideal final breast size. In only a small percentage of women who are large breasted, with ptosis and elevated BMI, can a reconstructed breast of appropriate volume and proportion be constructed in a single stage.^8^ We have previously shown that second-stage implant placement^9^ or lipotransfer^10^ can enhance the final result but carry with them the risks of a prosthetic and the more than likely need for additional fat-grafting surgeries, respectively. These approaches, at a minimum, require 2 surgical procedures.

We sought a solution to this problem by looking for a simple, safe autologous technique that could be performed at the same time as the Goldilocks mastectomy. The LICAP flap in combination with the Wise pattern has not been previously described in reconstructive breast cancer surgery, but is well described in the MWL patient.^8^ The MWL patients often have deflated, ptotic breasts, and excess lateral chest wall subcutaneous tissues very akin to our high-risk, obese Goldilocks patients. The donor site morbidity associated with the LICAP flap is minimal and does not have the same wound breakdown rate as a latissimus flap where the skin is often undermined, and the muscle mobilized.^9^

In our experience, there is no obvious association between obesity and LICAP flap failure as these are well established abdominally based autologous reconstructions.^6^ The dissection does not require microsurgical skills, as perforators are reliably located within 5 cm of the anterior border of the latissimus at the inframammary fold.^7^ The donor site is abundant in obese patients, and they are pleased to have this additional body contouring performed to help supplement their breast volume. The flap easily reaches the breast meridian and the most medial portions of the breast footprint. The LICAP donor site is an area of excess tissue typically left in place after a traditional mastectomy that is bothersome to many women. Although we did not suffer any complications with placing these morbidly obese patients prone to raise their flaps, one should certainly inform patients of the cardiopulmonary risk associated with this maneuver. We believe this approach should be selectively applied to those patients who refuse an implant and or abdominal flap and demand a single-stage reconstruction. This strategy is most appropriate in the obese patient population where traditional approaches have known high rates of complications including complete reconstructive failure that was not seen here.

## CONCLUSIONS

The Goldilocks mastectomy is a safe reconstructive strategy in patients who are at high risk for complications after traditional implant-based or autologous approaches after mastectomy surgery. Unfortunately, the Goldilocks mastectomy does not often allow for a definitive reconstruction in a single stage. We have found that addition of the LICAP flap to the Goldilocks reconstruction is a reliable and safe strategy which can provide many women enough supplemental volume to complete their autologous reconstruction in a single surgery. This strategy is best suited to the obese and or ptotic patients who are precisely the group of patients at highest risk for complications after more traditional reconstructive approaches.

## Supplementary Material

## References

[1] Richardson H Ma G The Goldilocks mastectomy. Int J Surg. 2012;10:522–526. 22892093 10.1016/j.ijsu.2012.08.003

[2] Fischer JP Cleveland EC Nelson JA Breast reconstruction in the morbidly obese patient: assessment of 30-day complications using the 2005 to 2010 National Surgical Quality Improvement Program data sets. Plast Reconstr Surg. 2013;132:750–761. 24076667 10.1097/PRS.0b013e31829fe33c

[3] Lin KY Johns FR Gibson J An outcome study of breast reconstruction: presurgical identification of risk factors for complications. Ann Surg Oncol. 2001;8:586–591. 11508620 10.1007/s10434-001-0586-3

[4] McCarthy CM Mehrara BJ Riedel E Predicting complications following expander/implant breast reconstruction: an outcomes analysis based on preoperative clinical risk. Plast Reconstr Surg. 2008;121:1886–1892. 18520873 10.1097/PRS.0b013e31817151c4

[5] Hamdi M Spano A Van Landuyt K The lateral intercostal artery perforators: anatomical study and clinical application in breast surgery. Plast Reconstr Surg. 2008;121:389–396. 18300954 10.1097/01.prs.0000298317.65296.cf

[6] Kwei S Borud LJ Lee BT Mastopexy with autologous augmentation after massive weight loss: the intercostal artery perforator (ICAP) flap. Ann Plast Surg. 2006;57:361–365. 16998323 10.1097/01.sap.0000222569.59581.d9

[7] Hakakian CS Lockhart RA Kulber DA Lateral intercostal artery perforator flap in breast reconstruction: a simplified pedicle permits an expanded role. Ann Plast Surg. 2016;76 (Suppl 3):S184–S190. 26914351 10.1097/SAP.0000000000000752

[8] Schwartz JC Skowronski PP Total single-stage autologous breast reconstruction with free nipple grafts. Plast Reconstr Surg Glob Open. 2015;3:e587. 26894012 10.1097/GOX.0000000000000563 PMC4727696

[9] Schwartz JC Goldilocks mastectomy: a safe bridge to implant-based breast reconstruction in the morbidly obese. Plast Reconstr Surg Glob Open. 2017;5:e1398. 28740796 10.1097/GOX.0000000000001398 PMC5505857

[10] Schwartz JD Skowronksi PP Extending the indications for autologous breast reconstruction using a two-stage modified goldilocks procedure: a case report. Breast J. 2017;23:344–347. 27914123 10.1111/tbj.12737

