# Supplementary figures and images for: Toward a More Definitive Goldilocks Mastectomy: Simultaneous Addition of the Lateral Intercostal Perforator Flap
Source: Plast Reconstr Surg Glob Open. 2019 Mar 13;7(3):e2132. doi: 10.1097/GOX.0000000000002132 (PMC6467631; doi:10.1097/GOX.0000000000002132)

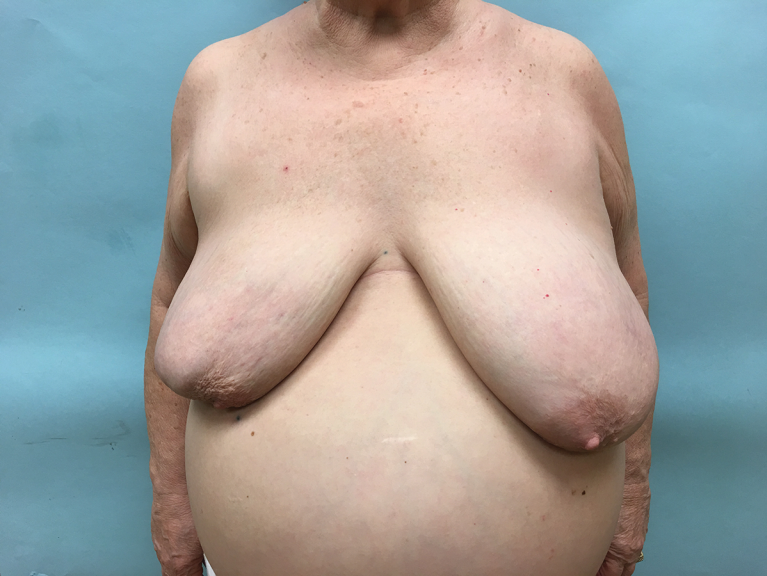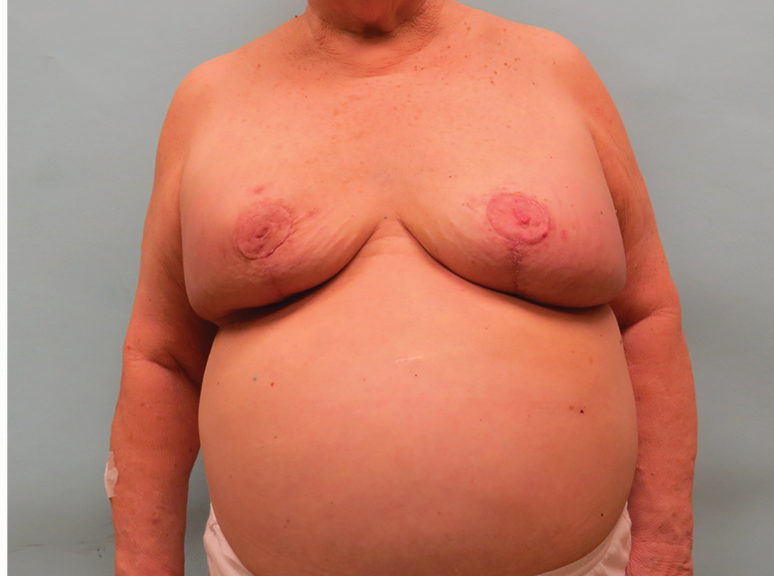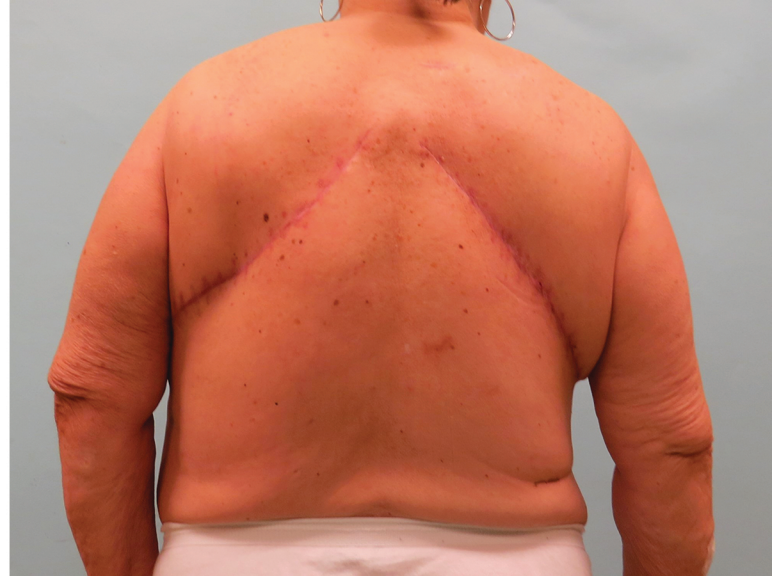

Supplement: Supplementary file 1 [file gox-7-e2132-s001.pdf]

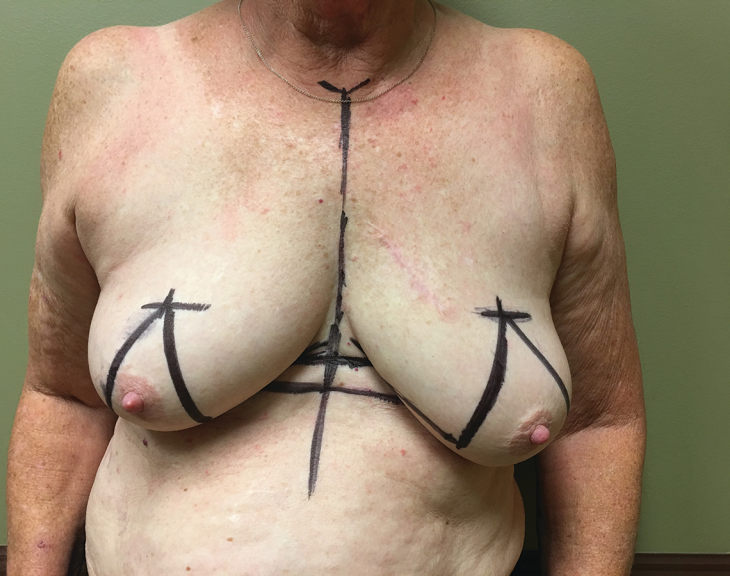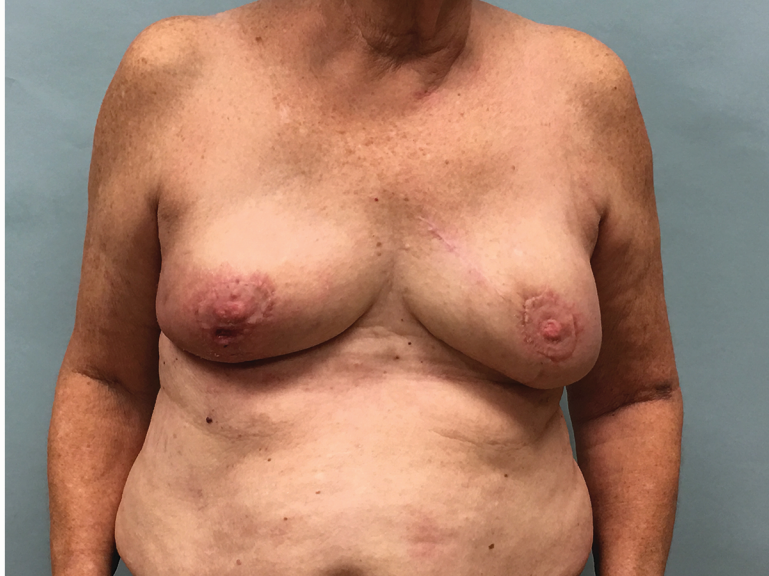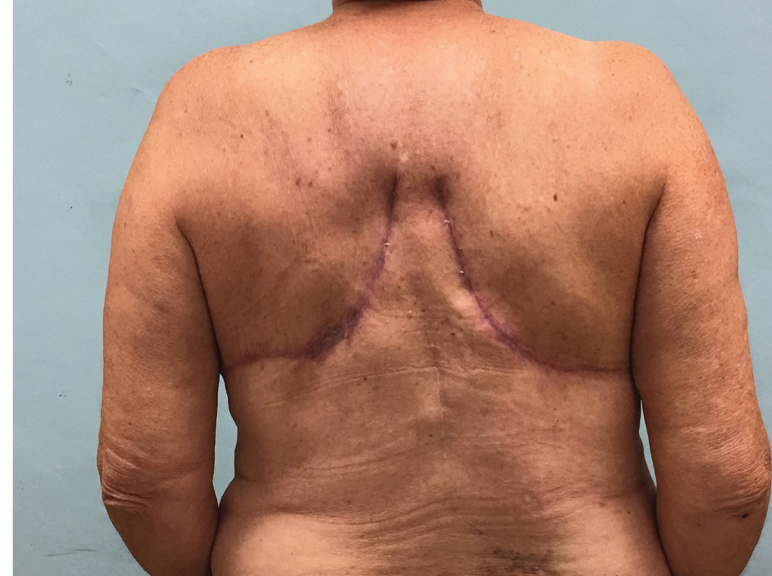

Supplement: Supplementary file 2 [file gox-7-e2132-s002.pdf]
